# Supplementary material for: Marker for kidney fibrosis is associated with inflammation and deterioration of kidney function in people with type 2 diabetes and microalbuminuria
Source: PLoS One. 2023 Mar 17;18(3):e0283296. doi: 10.1371/journal.pone.0283296 (PMC10022760; doi:10.1371/journal.pone.0283296)
Supplement: S1 Fig — (A) Distribution of sC3M (B) Distribution of uC3M (C) correlation analysis between sC3M and uC3M (D) correlation analysis between eGFR and sC3M and (E) correlation analysis between sC3M and UAER. (DOCX) [file pone.0283296.s001.docx]

**S1 Fig. Distribution and correlations.**


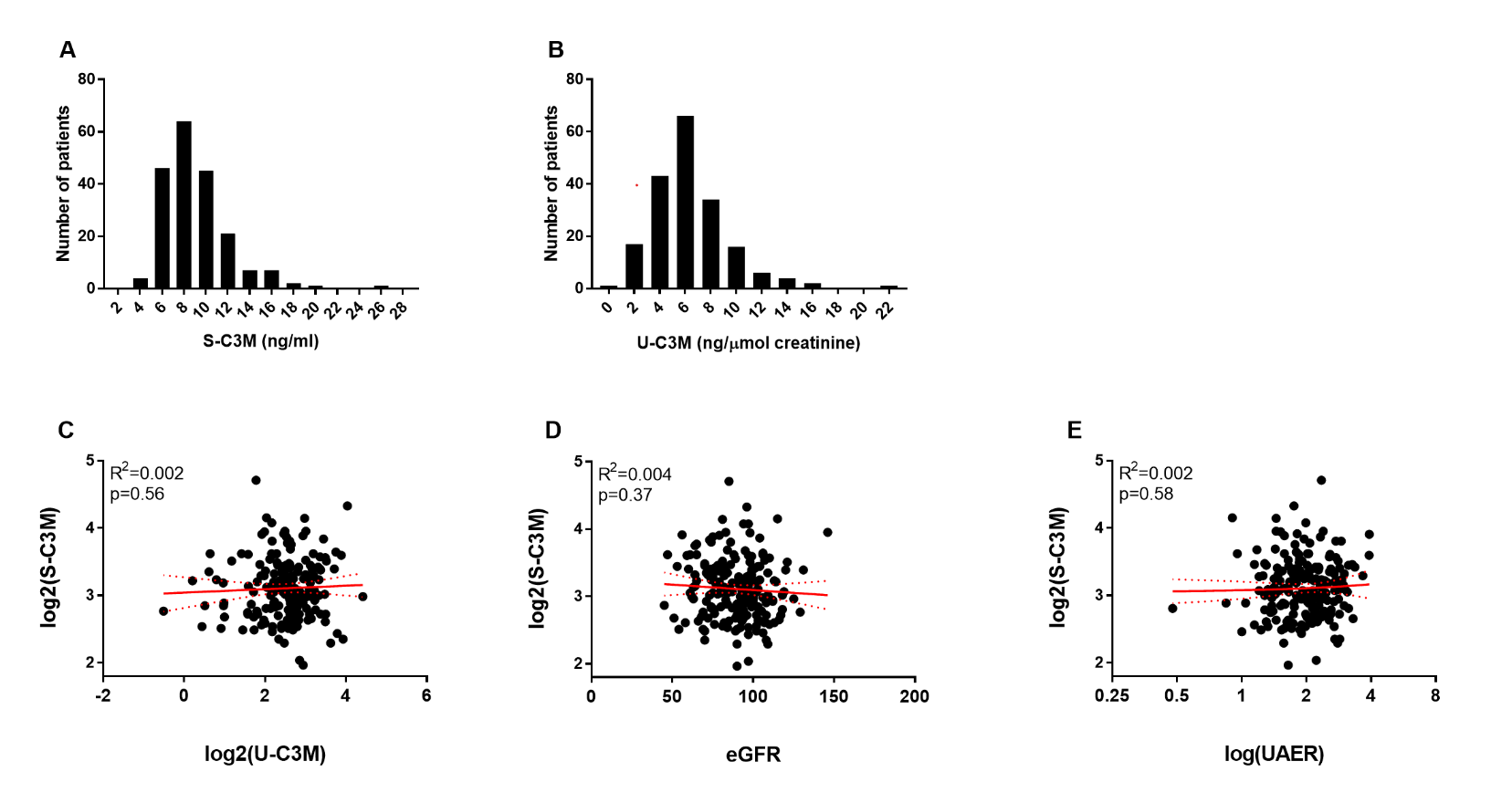


(A) Distribution of sC3M (B) Distribution of uC3M (C) correlation analysis between sC3M and uC3M (D) correlation analysis between eGFR and sC3M and (E) correlation analysis between sC3M and UAER.
