# Supplementary material for: Marker for kidney fibrosis is associated with inflammation and deterioration of kidney function in people with type 2 diabetes and microalbuminuria
Source: PLoS One. 2023 Mar 17;18(3):e0283296. doi: 10.1371/journal.pone.0283296 (PMC10022760; doi:10.1371/journal.pone.0283296)
Supplement: S1 Table — Data are expressed as mean ± SD, median (interquartile range) or number (%) as appropriate. eGFR: estimated glomerular filtration rate. HbA1c: glycosylated hemoglobin; RAAS: renin-angiotensin-aldosterone-system. P-value for trend across tertiles of uC3M. (DOCX) [file pone.0283296.s002.docx]

**S1 Table. Baseline characteristics for urine C3M tertiles**

| **Characteristic** | **All**  **n=198** | **Tertile 1**  **n=64** | **Tertile 2**  **n=64** | **Tertile 3**  **n=64** | **P-value** |
| --- | --- | --- | --- | --- | --- |
| **uC3M (ng/mol)** | 6.1 (4.5-7.7) | 3.9 (3.0- 4.5) | 6.1 (5.7- 6.5) | 8.7 (7.7- 10.3) | <0.001 |
| **Male, n (%)** | 149 (75) | 52 (81.2) | 52 (81.2) | 41 (64.1) | 0.033 |
| **Age (years)** | 58.6 ± 8.7 | 60.83 ± 7.89 | 58.70 ± 8.21 | 56.48 ±9.78 | 0.020 |
| **Known duration of diabetes (years)** | 12.7 ± 7.4 | 15.16 ± 7.28 | 11.39 ± 8.00 | 11.32 ± 6.41 | 0.003 |
| **Body mass index (kg/m2)** | 32.5 ± 5.8 | 32.88 ± 5.82 | 32.11 ± 4.58 | 32.82 ± 6.90 | 0.707 |
| **HbA1c (%)** | 7.9 ± 1.3 | 7.61 ± 1.22 | 7.65 ± 1.14 | 8.26 ± 1.54 | 0.008 |
| **HbA1c (mmol/mol)** | 62.3 ± 14.8 | 59.64 ± 13.32 | 60.11 ± 12.41 | 66.77 ± 16.85 | 0.008 |
| **Urinary albumin excretion rate (mg/24-h)** | 102 (39-229) | 87 (33-209) | 110 (41-251) | 110 [62 – 282) | 0.222 |
| **P-creatinine (μmol/L)** | 76.4 ± 18.4 | 85.73 ± 19.94 | 78.69 ± 14.99 | 65.66 ± 14.38 | <0.001 |
| **eGFR (ml/min/1.73m2)** | 90 ± 17 | 81.02 ± 16.98 | 88.00 ± 15.10 | 98.70 ± 16.12 | <0.001 |
| **LDL cholesterol (mmol/L)** | 1.9 ± 0.8 | 2.05 ± 0.89 | 1.68 ± 0.59 | 1.83 ± 0.81 | 0.026 |
| **Systolic blood pressure (mmHg)** | 130 ± 17 | 130.41 ± 18.49 | 128.53 ± 14.19 | 131.70 ± 19.05 | 0.585 |
| **Diastolic blood pressure (mmHg)** | 75 ± 9 | 72.61 ± 10.53 | 75.99 ± 11.97 | 75.54 ± 11.03 | 0.185 |
| **Current smoker, n (%)** | 59 (30) | 16 (25.0) | 17 (26.6) | 23 (35.9) | 0.338 |
| **Treatment with** |  |  |  |  |  |
| **Oral antidiabetic, n (%)** | 169 (85) | 49 (76.6) | 58 (90.6) | 56 (87.5) | 0.066 |
| **Insulin, n (%)** | 122 (62) | 38 (59.4) | 38 (59.4) | 43 (67.2) | 0.575 |
| **Antihypertensive drugs, n (%)** | 196 (99) | 64 (100.0) | 63 (98.4) | 64 (100.0) | 0.366 |
| **RAAS blockade, n (%)** | 194 (98) | 63 (98.4) | 62 (96.9) | 64 (100.0) | 0.362 |
| **Statin, n (%)** | 187 (94) | 57 (89.1) | 62 (96.9) | 63 (98.4) | 0.038 |
| **Aspirin, n (%)** | 182 (92) | 56 (87.5) | 61 (95.3) | 59 (92.2) | 0.274 |
